# Transition from the Nanoscale to Bulk in the Nonequilibrium Optical Response of Laser-Dressed Materials

**DOI:** 10.1021/acs.jpclett.5c02710

**Published:** 2025-12-29

**Authors:** Vishal Tiwari, Luis Sierra-Ossa, Pawel Wojcik, Ignacio Franco

**Affiliations:** † Department of Chemistry, 6927 University of Rochester, Rochester, New York 14627, United States; ‡ Department of Chemistry, 3270 Northwestern University, Evanston, Illinois 60208, United States; ¶ Department of Chemistry and Biochemistry, 7823 Florida State University, Tallahassee, Florida 32306, United States; § Department of Physics and Astronomy, University of Rochester, Rochester, New York 14627, United States; ∥ Institute of Optics, University of Rochester, Rochester, New York 14627, United States

## Abstract

Understanding how the behavior of materials transitions from the nanoscale to bulk highlights properties where the finite size of matter matters. To date, such studies have focused on materials at or near thermal equilibrium, while this transition for strongly driven nonequilibrium systems is not understood. Here we investigate for the first time this transition for laser-dressed Floquet-engineered materials where resonant and nonresonant light is used to drive matter out of thermal equilibrium, creating an effective nonequilibrium material with properties that can be very different from those of pristine matter and that can be triggered on demand. As an archetypical example, we computationally characterize the linear optical absorption of laser-dressed *trans*-polyacetylene as a function of chain length, and also in bulk, using first-principle Hamiltonians and a recently proposed theory for the nonequilibrium optical response. The computations reveal nonequilibrium absorption sidebands that converge to equivalent features for bulk as the size of the system is increased, in a manner akin to near-equilibrium behavior. The computations also reveal nonequilibrium low-frequency features that emerge because of hybridization of Floquet states that show a persistent dependence on system size. We further demonstrate how resonant driving can be used to transform the isolated absorption peaks of a nanomaterial into broad bulk-like features. Overall, this work characterizes the structure–function relations in Floquet-engineered materials.

## Graphical abstract

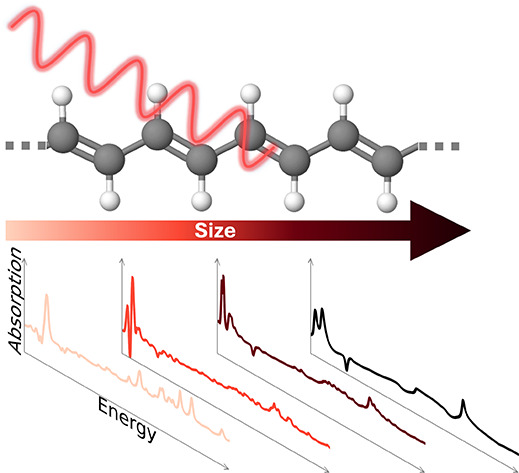

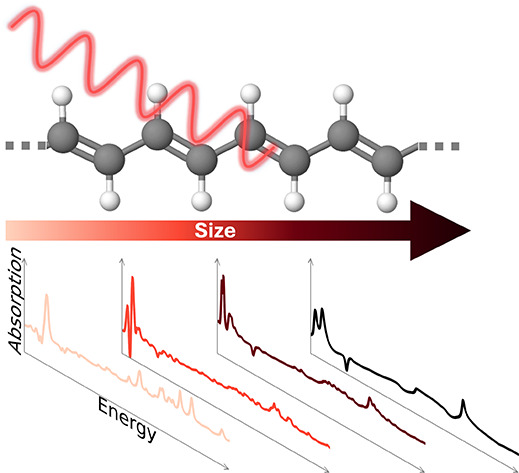

Strong light–matter interactions provide a versatile and powerful control strategy to transiently modify the physicochemical properties of matter. This direction is referred to as Floquet engineering^1–3^ when continuous wave time-periodic lasers are used to create nonequilibrium laser-dressed materials. Floquet engineering has been demonstrated to be useful in band structure engineering,^4–8^ controlling exciton dynamics,^9–11^ energy^12^ and electron^13^ transfer, and inducing chirality.^14,15^

At the foundation of Floquet engineering is the Floquet theorem,^16^ which is useful in solving the time-dependent Schrödinger equation (TDSE) $i\hbar \frac{d}{dt}\left|\psi \left(t\right)\right\rangle =Ĥ\left(t\right)\left|\psi \left(t\right)\right\rangle$ governed by the *T*-periodic Hamiltonian Ĥ(t+T)=Ĥ(t) . According to the Floquet theorem, the solutions to the TDSE are of the form $\left|\psi_{\alpha }\left(t\right)\right\rangle =e^{-iE_{\alpha }t/\hbar }\left|\phi_{\alpha }\left(t\right)\right\rangle$ , where the Floquet mode |ϕ_α_(*t*)⟩ = |ϕ_α_(*t* + *T*)⟩ is *T*-periodic. The Floquet modes {|ϕ_α_(*t*)⟩} and the quasienergies {*E*_α_} are obtained by solving an eigenvalue problem $\left(Ĥ\left(t\right)-i\hbar \frac{d}{dt}\right)\left|\phi \left(t\right)\right\rangle =E_{\alpha }\left|\phi \left(t\right)\right\rangle$ in an extended Hilbert space called Sambe space.^17^ This space is the tensor product of the regular Hilbert space and the space spanned by all *T*-periodic functions with basis {*e* ^*in*Ω*t*^}, where *n* are integers and Ω = 2π/*T*, which is akin to matter in the presence of quantum light. Expressed on the Floquet basis, the most general solution to the TDSE is |ψ­(*t*)⟩ = *∑*_α_ *c*_α_|ψ_α_(*t*)⟩, where {*c*_α_} are time-independent coefficients. In this way, the Floquet theory maps the quantum dynamics of a periodically driven system into a quasistationary problem in Sambe space.

A growing body of evidence is demonstrating that the Floquet modes are the natural basis to investigate the physical properties of laser-dressed matter.^18–22^ Their utility is that the properties of nonequilibrium matter can be understood through the quasistationary populations (|*c*_α_|^2^) of the Floquet states. Further, the Floquet picture remains useful even when the driving is done with ultrafast laser pulses, making the insights from Floquet engineering of broader applicability.^8,23,24^

Establishing structure–function relations such as the size-dependence of the optical absorption is a key aspect of material design. Such study of structure–function relations^25,26^ has led to advances in nanotechnology with applications, for instance, in understanding biological systems,^27,28^ designing materials with desired photophysical properties,^29^ controlling catalytic activity of reactions,^30,31^ and designing nanomaterial–microorganism hybrid systems for solar energy harvesting.^32^ For near-equilibrium matter, it is well understood how the physical properties of finite nanomaterials converge to its bulk limit as the size of the system is increased (see examples in ref 33). Such studies are important for identifying when the size of nanomaterials matters and to understand how its physical properties transition from being governed by discrete, molecular-like to continuous, band-like, electronic structure.

Despite growing interest in Floquet engineering, investigating the size-dependence of laser-dressed materials remains an open challenge. Previous studies have investigated the transition of the nonlinear optical response from the nanoscale to bulk in the presence of static electric fields^34,35^ and the efficiency of high harmonic generation for nanomaterials of increasing size.^36^ However, it remains unclear whether size thresholds that signal the transition from nano to bulk identified at equilibrium^37^ persist in laser-dressed nonequilibrium systems, where the Floquet modes, rather than pristine material states, determine the dynamical symmetries^20^ and physicochemical properties. Addressing this knowledge gap requires a detailed characterization of how the response properties of Floquet-engineered matter evolve with the system size.

Here we investigate the structure–function relations of laser-dressed matter. In particular, we study for the first time how the optical absorption properties of laser-dressed matter change as the size of the system is increased from the nanoscale up to its bulk limit. At a technical level, addressing this important problem requires a method to compute the nonequilibrium optical absorption of laser-dressed matter with first-principle Hamiltonians that is invariant to the light–matter interaction gauge. The issue in gauge invariance emerges because computational convenience requires different choices of gauges in nanoscale and bulk material. For finite-sized matter, the length gauge is preferred, where the light–matter interaction operator is defined through the position operator. By contrast, for solids the length gauge becomes inconvenient due to the ill-defined nature of the position operator^38,39^ and the velocity gauge emerges as a more convenient choice^40–42^ as it allows invoking the Bloch theorem even in presence of external fields. However, the computation with first-principles material Hamiltonians based on the velocity gauge are unfeasible due to the requirement of a large number of bands for convergence and because the truncation of Hilbert space can lead to breaking of gauge invariance.^43–48^ This leads to inconsistencies in the computation of the physical properties for laser-dressed nanoscale materials with those of their bulk counterpart.

In this Letter, we overcome this issue and finally demonstrate the transition of the nonequilibrium optical absorption from the nanoscale to bulk of a realistic laser-dressed material. Recent theoretical advances now enable this characterization for nanomaterials^49^ and solids^22^ using first-principles density functional theory (DFT) Hamiltonians.^48^ The electronic structure of the nanomaterial is described through Kohn–Sham molecular orbitals while that of the solid is described through Bloch bands. Particularly for laser-dressed solids, consistent comparison of the computations with nanomaterial are now feasible due to the development of (i) a Floquet-based approach to efficiently compute the full frequency dependence of the nonequilibrium optical response, (ii) a truncated velocity gauge for the light–matter interactions that enables simulations with first-principle Hamiltonians and that overcomes the convergence issues of the velocity gauge with the number of bands, and (iii) the massively parallel computational implementation of the theory.^50^ These approaches exactly capture the influence of a driving laser field of arbitrary frequency and intensity through the Floquet formalism and provide a strategy to compute the nonequilibrium optical response to weak probing light across the electromagnetic spectrum.

Figure 1 summarizes the emerging electronic structure and optical absorption phenomenology for laser-dressed materials as a function of the intensity of driving laser as identified in our previous studies.^22,49^ For equilibrium matter (I), optical transitions from the valence (red) to the conduction (blue) band yield an absorption spectrum. As the system is driven by nonresonant light of photon energy *ℏ*Ω, the laser-dressing creates Floquet replicas of the valence and conduction band that are separated from each other by integer multiples of *ℏ*Ω. Optically probing these Floquet replicas leads (II) to below-band gap absorption. In addition, the laser-dressing shifts the energy of the pristine valence and conduction bands, resulting in a net blue-shift of band edge, akin to the Stark and Bloch–Seigert shift.^51,52^ Further increasing the driving laser intensity opens optical transitions to additional Floquet replicas. Transitions that happen among these replicas lead to (III) absorption sidebands, optical features that are separated by integer multiples of *ℏ*Ω in the absorption spectrum. At even higher laser intensities, the Floquet replicas of the valence and conduction band can energetically overlap and hybridize, thus opening an energy gap. Probing transition between these hybrid bands (purple) leads to (IV) intense absorption and stimulated emissions in the mid-infrared frequencies, which we refer to as low-frequency transitions.

**Figure 1:**
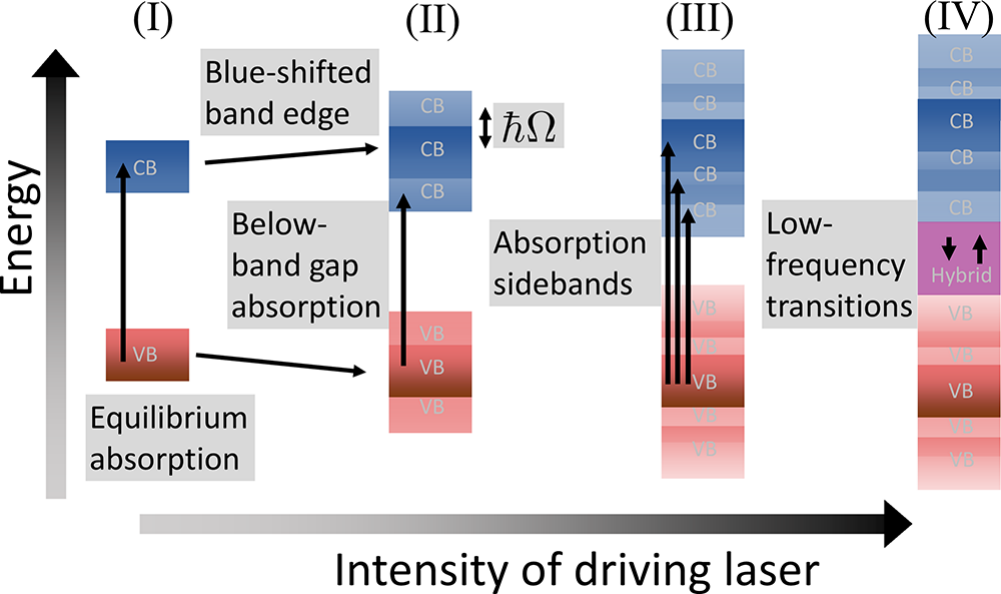
Diagrammatic representation of various phenomena that occur in the optical absorption of laser-dressed solids. Dressing materials with time-periodic light creates Floquet replicas of the valence (red) and conduction (blue) bands that are separated from each other by integer multiples of the drive photon energy *ℏ*Ω. Optically probing this laser-dressed band structure induces transitions among these Floquet replicas (black vertical arrows). Below-band gap absorption is due to transitions between replicas separated by energy lower than the band-edge energy. Absorption sidebands arise because of this replicated structure. Overlapping replicas can hybridize, leading to low-frequency transitions.

The fundamental question that we address here is how the effective electronic structure of laser-dressed materials transitions from the nanoscale to bulk, as reflected in the optical properties of matter. As an illustrative model, we consider *trans*-polyacetylene (*t*PA) oligomers as it is a prototypical example of a semiconducting organic material.^8,53–57^ Throughout, we consider a drive-probe physical situation in which a continuous wave laser of arbitrary amplitude (*E*_d_) and frequency (Ω) creates the nonequilibrium material. The effective absorption properties of this laser-dressed material are then probed by a weak continuous wave laser source with amplitude *E*_p_ and frequency ω. The drive and probe laser are taken to be linearly polarized along the chain length. The spectra per unit volume for finite chains is obtained by using the method in ref 49, while the one for bulk in refs 48 and 58 . To describe the *t*PA we employ a first-principles Hamiltonian using DFT followed by Wannier interpolation that facilitates the computation of the nonequilibrium optical absorption. Details of the theories to capture the optical absorption of laser-dressed matter and the electronic structure computation are included in Methods.

Consider first the optical absorption of equilibrium matter. Figure 2 shows the computed equilibrium absorption spectra. That is, the net absorption as a function of probe photon energy (*ℏω*), for the nanomaterial (red lines) with *N* = 20, 40, 60 and 80 unit cells (corresponding to chain length of 5, 10, 15, and 20 nm, respectively) and for bulk (blue line). The spectra are shifted along the *y*-axis for the sake of clarity.

**Figure 2:**
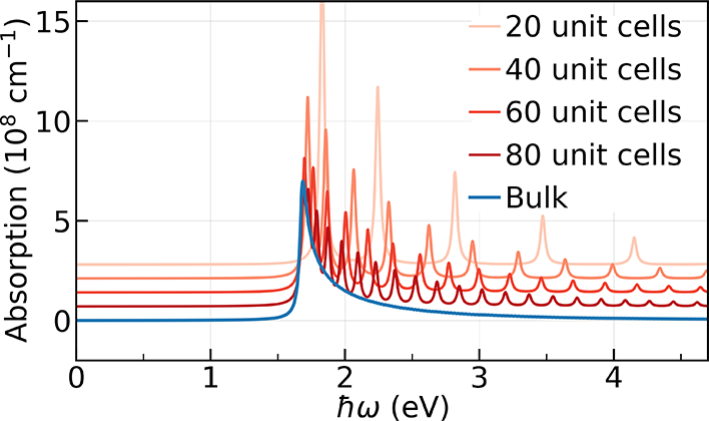
Optical absorption spectrum per unit volume of *trans*-polyacetylene (*t*PA) as a function of system size with 20, 40, 60 and 80 unit cells (red) and bulk (blue). Spectra are shifted vertically for clarity. The spectra for the nanomaterial converges to that of bulk upon increasing the chain length.

As expected, the absorption spectra of finite-length chains converge toward the bulk limit upon increasing chain length. The bulk spectra exhibits the main absorption peak at *ℏω*_0_ = 1.67 eV corresponding to the band-edge transition at Γ point (reciprocal vector *k* = 0 in the Brillouin zone) and decays as $\propto 1/\sqrt{\hbar \omega -\hbar \omega_{0}}$ due to its one-dimensional structure. This band-edge transition is already properly captured by the highest occupied molecular orbital (HOMO) to lowest unoccupied molecular orbital (LUMO) transition in the 40-cell *t*PA. Beyond the band-edge transition, the 80-cell *t*PA shows oscillatory features that closely follow the spectrum for bulk *t*PA. In contrast, the 20-cell *t*PA, the shortest chain considered, displays fewer peaks because of its lower density of states. As the chain length increases, these peaks progressively condense and lead to the continuous spectrum characteristic of the bulk.

Consider now the case of nonequilibrium matter. Figure 3 shows spectra for *t*PA oligomers driven with a laser of amplitude *E*_d_ = 0.5 V/nm (in Figure 3a–d) and *E*_d_ = 1 V/nm (in Figure 3e–h) for varying *ℏ*Ω. This corresponds to an intermediate regime of light–matter interaction where the effects of the driving laser is nonperturbative but below the limit of dielectric breakdown. The spectra for nanomaterials with 20, 40, 60 and 80 unit cells (red lines) are shifted along the *y*-axis for clarity with respect to the spectra for bulk (blue lines). To highlight the different optical features in each plot, green arrows mark the position of the bulk’s main absorption peak due to transition at the Γ point. These transitions are blue-shifted in the presence of laser-dressing from 1.67 eV (*ℏω*_0_) as schematically shown in Figure 1 as the field-free Bloch bands show AC-Stark shift and Bloch–Siegert shift due to the formation of Floquet replicas.^9,59^ In turn, the purple arrows mark the one-photon absorption sideband that is *ℏ*Ω away from the main absorption peak as it arises due to transition among the Bloch band and the one-photon Floquet replica (see Figure 1). The visibility of this absorption sideband is dependent on the driving laser parameters. Here, it is more salient for driving amplitudes *E*_d_ = 1.0 V/nm and near-resonant frequencies (*ℏ*Ω = 1.3, 1.5 eV) compared to other sets of parameters.

**Figure 3:**
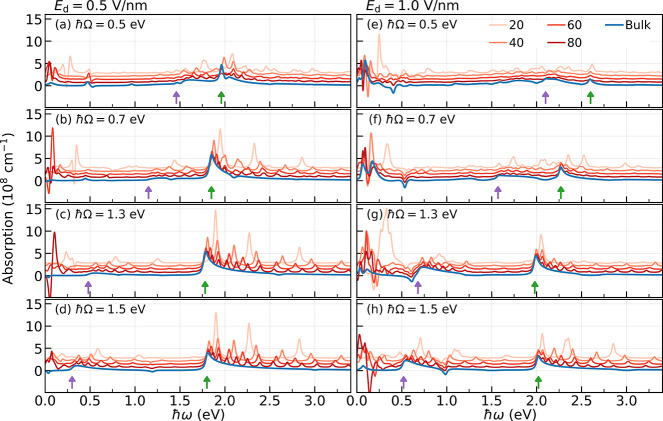
Nonequilibrium optical absorption spectrum per unit volume from the nanoscale to bulk for *t*PA oligomers. The plot shows the optical absorption of chains with 20, 40, 60 and 80 unit cells (red) and bulk (blue) that are driven by a laser with amplitude (a–d) *E*_d_ = 0.5 V/nm and (e–h) *E*_d_ = 1.0 V/nm and of varying laser photon energy *ℏ*Ω. The spectra for nanomaterials are shifted vertically for clarity. The green arrows signal the bulk’s absorption feature at the Γ point (that is at *ℏω* = *E*_Γ_), and purple arrows indicate its one-photon absorption sideband (at *ℏω* = *E*_Γ_ – *ℏ*Ω). Note the nanomaterial spectra converge to that of the bulk for the absorption sideband and the main absorption features, but such convergence is not apparent in the low-frequency region (*ℏω* < 0.3 eV).

Figure 3 shows that under nonequilibrium situations the main absorption features and sidebands of laser-dressed *t*PA chains do converge toward those of bulk as the chain length increases (for the range of driving laser parameters considered). For instance, the 40-cell *t*PA already reproduces the peak positions of the arrow-marked bulk features across the whole range of driving laser conditions, consistent with its equilibrium behavior (Figure 2). In turn, the 60- and 80-cell chains even match the corresponding absorption intensities per unit volume. The spectra for 20-cell *t*PA show isolated peaks throughout that do not align with the bulk spectra as expected due to its difference in electronic structure from longer chains, which is also evident at equilibrium in Figure 2.

However, Figure 3 also reveals differences in the convergence behavior from the nanoscale to the bulk with respect to equilibrium. The spectra in the mid-infrared frequency region (*ℏω* ∈ [0, 0.3] eV) show absorption and stimulated emission features that are strongly dependent on the system size for all the driving laser conditions. Even for long chain size with 80 unit cells, the spectra for the nanomaterial does not converge to the respective features for bulk and presumably will require much longer chains to replicate the features for the bulk. These persistent differences highlight the impact of laser dressing on the nanoscale-to-bulk transition of the optical response.

The strong dependence on chain length for the low-frequency features seen in Figure 3 arises because the net effect is due to competing contributions that lead to a strong dependence on the effective density of states. The low-frequency transitions in laser-dressed matter arise from the hybridization of Floquet states for both nanomaterials^49^ and bulk^22^ (see Figure 1). Each hybridization opens a gap that introduces a low-frequency transition into the spectra. For the nanomaterial and also for bulk, many of the Floquet states undergo hybridization and the net signal is the result of competing transitions that lead to absorption or stimulated emission that do not completely cancel out (see Figure S1 in the Supporting Information^60^ for spectra detailing the individual transition lines in both nanomaterial and bulk that demonstrate this effect). For this reason, these transitions are highly sensitive to the effective density of states and energy level distribution of the material, even for the same driving laser conditions.

The dressing of the material through strong fields also opens the possibility of engineering the effective density of states that is evident in the optical response of nanomaterials. To demonstrate this, Figure 4 compares the optical absorption spectrum of an 8 unit cell *t*PA chain to that of bulk in the absence and in the presence of driving laser, respectively. As seen in Figure 4a, the spectrum for the field-free 8-cell *t*PA shows two isolated absorption peaks, one at 2.26 eV due to HOMO to LUMO transition and another one at 3.64 eV due to the HOMO–1 to LUMO+1 transition. In turn, the bulk band edge transition is at 1.67 eV. As the material is driven by a laser with amplitude *E*_d_ = 0.5 V/nm and photon energy *ℏ*Ω = 0.7 eV, the absorption spectra of the 8-cell *t*PA (Figure 4b) now shows a multipeaked broad absorption feature centered at 2.25 and 3.64 eV due to the emergence of multiple absorption peaks around that energy. We find that this multipeaked feature for the 8-cell *t*PA is specific to *ℏ*Ω ≈ 0.7 eV.

**Figure 4:**
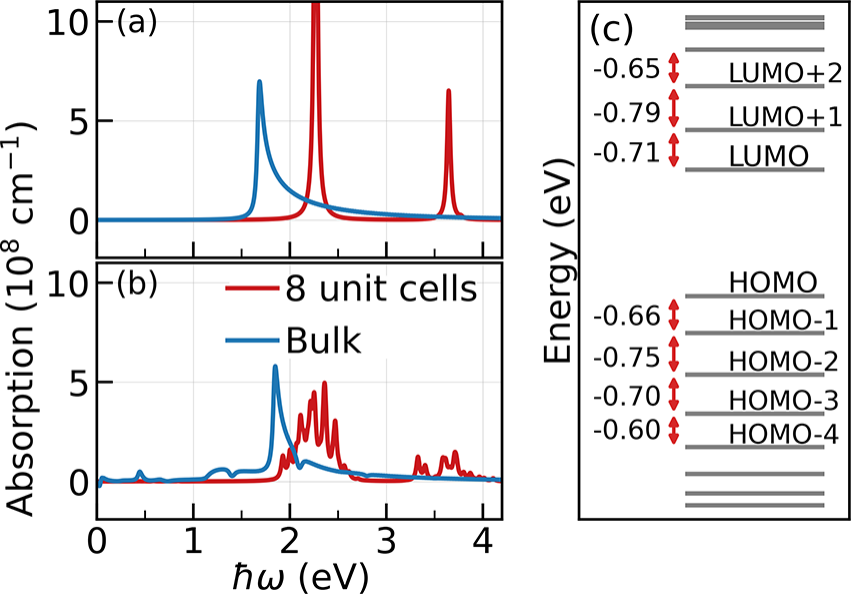
Transforming the effective density of states from molecular-like to band-like in nanoscale using resonant driving. Optical absorption spectrum of *t*PA with 8 unit cells (red) and bulk (blue) for (a) pristine matter and (b) in the presence of resonant driving with *E*_d_ = 0.5 V/nm and *ℏ*Ω = 0.7 eV. (c) Energy levels of 8 cell *t*PA in gray showing the values of the energy spacing. When the driving is near-resonant to the energy spacings between the neighboring levels to HOMO and LUMO, the isolated peaks in the spectra of a nanomaterial transform to multipeaked broad features indicating an increase in optically addressable density of states.

The multiple absorption peaks in Figure 4b arise due to transitions induced from the hybridization of Floquet replicas. The energy level structure for the pristine material is shown in Figure 4c Driving the 8-cell *t*PA with photon energy of *ℏ*Ω = 0.7 eV creates the −*ℏ*Ω Floquet replica of the LUMO+1 state that now energetically overlaps with the LUMO state (the gap between LUMO to LUMO+1 state is near-resonant to the laser as shown in Figure 4c) and hybridizes as the adjacent states are optically coupled. This results in two hybrid states that are now optically coupled to the HOMO, and optically probing them leads to two absorption peaks instead of the isolated absorption peak at equilibrium. Similar hybridization also occurs among the HOMO to HOMO–1 as the energy gap is also around 0.7 eV, leading to further splitting of the already split absorption peak. However, in this strongly driven system, multiple Floquet replicas of the higher-lying states can hybridize with LUMO (or the lower-lying states with HOMO) through the Autler–Townes^61^ effect even though they are not directly coupled, but they are coupled through the adjacent states. Optically probing these hybrid energy levels results in splitting of the HOMO-to-LUMO absorption peak into multiple peaks that coalesce into a broader absorption feature as seen in Figure 4b. The splitting is not symmetric in energy as the energy difference and coupling strengths of the adjacent levels is not equal. In Figure S2 in the Supporting Information^60^ we show that the Floquet states that lead to the transitions in the broad absorption feature have contributions from multiple pristine states due to this hybridization. Overall, the emergence of a multipeaked broad absorption feature signals an increase in the effective density of states in the nanomaterial due to resonant driving.

In conclusion, we characterized the transition from the nanoscale to bulk in the nonequilibrium optical response of laser-dressed materials using first-principles methods. Enabled by recent gauge-invariant theories for finite and extended systems,^48,49,58^ we computed the optical absorption spectra of laser-dressed *trans*-polyacetylene chains of varying length and compared them to the bulk limit. Our results reveal that laser dressing alters the convergence of spectral features with system size compared to that at equilibrium. While the main absorption peak and sidebands converge for chains with 40 unit cells, low-frequency features due to hybridization of the Floquet states depend strongly on system size, requiring longer chains due to sensitivity to the effective density of states. We further show that resonant driving can tune this effective density of states and induce bulk-like behavior in nanoscale systems.

Overall, our work establishes fundamental structure–function relations for laser-dressed nanomaterials and guides the design of Floquet-engineered nanostructures with tailored optical properties. Future prospects include investigating the nanoscale-to-bulk transition in two-dimensional materials^19,24,62–64^ for which Floquet states have been identified and in cases where size convergence only occurs in the mesoscale.^65,66^

## Methods

Here we summarize the theoretical developments outlined in refs 22, 48, and 49 used to obtain the optical absorption spectra of laser-dressed matter and the details of the electronic structure computation of *trans*-polyacetylene.

### Quantifying the Optical Response of Laser-Dressed Matter

For nonequilibrium matter, the net absorption of photons needs to be redefined because, in this regime, there is no stationary reference state and energy is no longer a conserved quantity. Thus, the increase of the energy of a system from a given reference state can no longer be used as a criterion for the optical absorption. In addition, the fluctuation–dissipation theorem^67^ and Green–Kubo^68,69^ relations, which form the basis of the near-equilibrium linear response theory,^70^ are no longer valid since the Hamiltonian in the presence of a driving laser breaks the time-translational symmetry.

To capture the optical absorption in laser-dressed matter,^22,48,49^ we compute the transition rate among laser-dressed states due to the interaction with a probe photon. The theory captures the driving laser exactly, while the effect of the probe is captured to first order in perturbation theory. This yields the transition rate^48,49^

1 $$I\left(\omega \right)\propto \iint_{t_{0}}^{t}dt_{1}dt_{2}C_{\mathrm{X̂},\mathrm{X̂}}\left(t_{1},t_{2}\right)\times Re\left[e^{-i\omega \left(t_{1}-t_{2}\right)}-e^{-i\omega \left(t_{1}+t_{2}\right)}\right]$$

where *t*_0_ is the preparation time. These transition rates are determined by a two-time correlation function $C_{\mathrm{X̂},\mathrm{X̂}}\left(t_{1},t_{2}\right)=Tr\left[\rho {\mathrm{X̂}}_{I}\left(t_{1}\right){\mathrm{X̂}}_{I}\left(t_{2}\right)\right]$ , where ρ is the density matrix and ${\mathrm{X̂}}_{I}\left(t\right)=U^{†}\left(t,t_{0}\right)\mathrm{X̂}U\left(t,t_{0}\right)$ is the light–matter coupling operator X̂ (for e.g. momentum or dipole) in the interaction picture of the laser-dressed Hamiltonian ${Ĥ}_{LD}\left(t\right)$ (sum of the material Hamiltonian and the interaction part with driving laser) with $U\left(t,t_{0}\right)=Te^{-i/\hbar \int_{t_{0}}^{t}H_{LD}\left(\tau \right)d\tau }$ being the time-ordered evolution operator for time *t*_0_ → *t*. The detailed derivation and exact expressions for eq 1 for finite systems are given in ref 49 and for bulk in ref 48. The two times *t*_1_ and *t*_2_ in the correlation function arise due to the breaking of time-translation symmetry of the system in the presence of the driving laser. In the absence of driving, time-translational symmetry is recovered and eq 1 reduces to the near-equilibrium response where the optical absorption emerges from a single time correlation function $\left(I_{eq}\left(\omega \right)\propto \int C_{\mathrm{X̂}}\left(\tau \right)e^{i\omega \tau }d\tau \right)$ .^70^

### Optical Response of Laser-Dressed Finite Nanomaterials

Reference 49 details how to compute the optical absorption spectrum of laser-dressed nanomaterial. In this case, the light–matter interaction is considered in the length gauge, where X̂→μ̂ , with $\mathrm{\mu ̂}=\mathrm{\mu ̂}\cdot \varepsilon_{p}$ being the projection of the material’s dipole operator μ̂ in the direction of the probe with polarization ***ε***_p_. The absorption coefficient *A*_n_(ω) per unit volume for laser-dressed nanomaterial, defined as the ratio of the power absorbed per unit volume to the incident flux of the probe field, is given by

2 $$A_{n}\left(\omega \right)=\frac{R_{n}\left(\omega \right)\hbar \omega }{VI_{0}}=\frac{\omega }{4\pi V\varepsilon_{0}cn_{r}}\underset{\lambda ,\lambda^{\prime }}{\sum }\underset{n}{\sum }|\mu_{\lambda^{\prime }\lambda }^{\left(n\right)}{|}^{2}P_{\lambda \lambda^{\prime }}\times [\delta \left(E_{\lambda^{\prime }\lambda }+n\hbar \Omega -\hbar \omega \right)-\delta \left(E_{\lambda^{\prime }\lambda }+n\hbar \Omega +\hbar \omega \right)]$$

where *R*_*n*_(ω) is the rate of net absorption (eq 46 in ref 49), *V* is the total volume of the material, *I*_0_ = *ε*_0_|*E*_p_|^2^ *cn*_*r*_/2 is the incident intensity of the probe field with *ε*_0_ the vacuum electric permittivity, *c* the speed of light in vacuum, and *n*_*r*_ the real part of the refractive index of the material. Here, *E*_λ′λ_ = *E*_λ′_ – *E*_λ_ where *E*_λ′_ (or *E*_λ_) is the corresponding quasienergy of the time-periodic Floquet mode |Φ_λ′_(*t*)⟩ (or |Φ_λ_(*t*)⟩). The quasienergies and the Floquet modes are obtained by solving the eigenvalue problem $\left(i\hbar \frac{\partial }{\partial t}-{Ĥ}_{LD}\left(t\right)\right)\left|\Phi_{a}\left(t\right)\right\rangle =E_{a}\left|\Phi_{a}\left(t\right)\right\rangle$ , where ${Ĥ}_{LD}\left(t\right)$ is the single-particle equivalent of the laser-dressed Hamiltonian. The quasienergies and corresponding Floquet modes are uniquely defined in a Floquet–Brillouin zone (FBZ); for example, the first FBZ is such that – *ℏ*Ω/2 ≤ *E*_λ_ < *ℏ*Ω/2.

In eq 2, the first term represents absorption, and the second term, stimulated emission. Here, $\mu_{\lambda^{\prime }\lambda }^{\left(n\right)}$ is the *n*-th Fourier component of the time-periodic transition dipole $\mu_{\lambda^{\prime }\lambda }=\left\langle \Phi_{\lambda^{\prime }}\left(t\right)\right|\mathrm{\mu ̂}\left|\Phi_{\lambda }\left(t\right)\right\rangle$ among the Floquet modes λ′, λ. The population factor *P*_*λλ*′_ ensures that the transitions happen from a filled state to an empty laser-dressed state.

Equation 2 shows that the optical response of laser-dressed nanomaterials emerges from transitions among the Floquet modes across multiple Floquet–Brillouin zones. The energy of a transition is given by the Bohr transition energy *E*_λ′λ_ + *nℏ*Ω representing the transition from λ → λ′ Floquet mode and the *nℏ*Ω term representing the *n*-FBZ difference. The transition dipole $\mu_{\lambda^{\prime }\lambda }^{\left(n\right)}$ (that is now dependent on the FBZ difference *n*) and population factor *P*_*λλ*′_ are also defined among the Floquet modes λ and λ′. In this nonequilibrium situation, the transition dipole and the population factors depend on the driving laser parameters instead of being inherent properties of the nanoscale material.

### Optical Response of Laser-Dressed Bulk Solids

To compute the optical absorption spectrum of laser-dressed crystals, we follow ref 48. For solids with space-periodic material Hamiltonian, the light–matter interaction is considered in the truncated velocity gauge.^46,48,71^ In this case, X̂→P̂ , where $\mathrm{P̂}=\mathrm{P̂}\cdot \varepsilon_{p}$ with P̂ the canonical momentum operator and ***ε***_p_ the probe laser polarization, but considered as a series of nested commutators to a given order, that is

3 $$\mathrm{P̂}\to \frac{m_{e}}{i\hbar }(\left[\varepsilon_{p}\cdot \mathrm{r̂},{Ĥ}_{0}\right]+\frac{1}{2!}\frac{eA_{d}\left(t\right)}{i\hbar }\left[\varepsilon_{p}\cdot \mathrm{r̂},\left[{\mathrm{\varepsilon ̂}}_{d}\cdot \mathrm{r̂},{Ĥ}_{0}\right]\right]+\frac{1}{2!}\frac{eA_{d}\left(t\right)}{i\hbar }\left[{\mathrm{\varepsilon ̂}}_{d}\cdot \mathrm{r̂},\left[{\mathrm{\varepsilon ̂}}_{p}\cdot \mathrm{r̂},{Ĥ}_{0}\right]\right]+\cdot \cdot \cdot )$$

Here, $A_{d}\left(t\right)=A_{d}\left(t\right){\mathrm{\varepsilon ̂}}_{d}$ is the vector potential of the drive laser with polarization vector ${\mathrm{\varepsilon ̂}}_{d}$ , r̂ the position operator, and ${Ĥ}_{0}$ the material’s Hamiltonian. For a drive-probe situation, the right side in eq 3 is exactly equal to the canonical momentum operator P̂ provided the Hilbert space is complete. However, any basis set truncation, as employed in any practical computation with first-principle material Hamiltonian, can violate this relation, leading to the replacement in eq 3.

The truncated velocity gauge provides an intermediate between the length and velocity light–matter gauge that is specifically useful when the Hamiltonian for the solid is constructed from first-principles. This is because it provides faster convergence of the computation with respect to the number of bands over the usual velocity gauge while still maintaining the space-periodicity of the Hamiltonian, unlike the length gauge. The truncated velocity gauge can be conveniently implemented using maximally localized Wannier functions and a generalized tight-binding model of the solid. This strategy also enables the exact comparison of the optical response of laser-dressed bulk with nanoscale matter by using the same level of electronic structure.

The formula for the absorption coefficient of laser-dressed bulk crystal^48^ in truncated velocity gauge is given by

4 $$A_{b}\left(\omega \right)=\frac{e^{2}\pi }{m_{e}^{2}\varepsilon_{0}cn_{r}V\omega }\underset{k}{\sum }\underset{\alpha ,\beta }{\sum }\underset{n}{\sum }\Lambda_{\alpha \beta k}|Z_{\alpha \beta k}^{\left(n\right)}{|}^{2}\times \left[\delta \left(E_{\alpha \beta k}+n\hbar \Omega -\hbar \omega \right)-\delta \left(E_{\alpha \beta k}+n\hbar \Omega +\hbar \omega \right)\right]$$

where **k** represents the reciprocal space vectors in the Brillouin zone, *m*_*e*_ is the mass of an electron, and *V* is the crystal’s volume. Here, *E*_*αβ* **k**_ = *E*_α**k**_ – *E*_β**k**_ with *E*_β**k**_ being the quasienergy corresponding to the Floquet–Bloch mode |Φ_β**k**_(*t*)⟩, a state that is space- and time-periodic. In turn, $Z_{\alpha \beta k}^{\left(n\right)}$ is the *n*th Fourier component of the time-periodic truncated momentum matrix element between the Floquet–Bloch mode α and β at crystal momentum **k** and Λ_*αβ* **k**_ is the corresponding population factor. The quasienergies {*E*_α**k**_} and Floquet–Bloch modes {|Φ_α**k**_(*t*)⟩} are obtained by solving the eigenvalue problem $\left(i\hbar \frac{\partial }{\partial t}-{Ĥ}_{k}\left(t\right)\right)\left|\Phi_{\alpha k}\left(t\right)\right\rangle =E_{\alpha k}\left|\Phi_{\alpha k}\left(t\right)\right\rangle$ in Sambe space,^17^ where ${Ĥ}_{k}\left(t\right)$ is the single-particle Hamiltonian form for the crystal Hamiltonian plus the driving laser interaction term.

Similar to the case of nanomaterials, the first term in eq 4 represents absorption, while the second represents stimulated emission. In this case, a transition event in the laser-dressed solid occurs from the Floquet–Bloch mode β → α across *n* FBZ with crystal momentum **k** (vertical in the reciprocal space) and transition energy *E*_α**k**_ – *E*_β**k**_ + *nℏ*Ω. The transition intensity is now determined by population factor Λ_*αβ* **k**_ and *n*-th Fourier component of the truncated momentum operator $Z_{\alpha \beta k}^{\left(n\right)}$ which are directly dependent on the driving laser parameters instead of being the inherent property of the crystal. Overall, in this case, eq 4 exemplifies that the Floquet–Bloch modes are the natural states to understand the optical absorption properties of laser-dressed solids as the transition is seen to emerge from them.

### Electronic Structure of *trans*-Polyacetylene

The calculation of the optical response of laser-dressed *trans*-polyacetylene oligomers, in nanoscale and bulk form, employs its first-principle material Hamiltonian in the tight-binding basis as constructed from maximally localized Wannier functions (MLWFs) .

For an isolated one-dimensional *trans*-polyacetylene chain, the parameters for generalized tight-binding Hamiltonian are obtained by performing the first-principles electronic structure computation based on density functional theory (DFT) using Quantum Espresso^72^ followed by Wannier interpolation of the DFT electronic structure using Wannier90.^73^ For DFT computations, we use an ultrasoft pseudopotential, local-density approximation (LDA) exchange–correlation functional, and a plane wave cutoff of 100 Ry. We use the bond length alteration of 1.34/1.54 and unit cell of length 2.5 Å for the geometry of an isolated *t*PA chain.^74^ We obtain a band gap of 1.67 eV at the Γ point (*k* = 0) in the BZ. For the Wannier interpolation, we choose the highest valence and lowest conduction band of *t*PA and interpolate for 100 *k*-points in the Brillouin zone. We retain up to three nearest-neighbor hopping parameters and intracell transition dipole matrix elements. For a chain with *N* unit cells, the model yields 2*N* eigenstates, of which the lowest *N* states are occupied. In the bulk limit, these MLWFs lead to a two band model with lower (or upper) band as valence (or conduction) band, and we use 1000 *k*-vectors to discretize the first Brillouin zone.

To obtain the optical absorption spectra, each transition line computed using eqs 2 and 4 is broadened using a Lorentzian function of full width at half maxima of 40 meV. To obtain the converged spectra, we employed 201 Floquet Fourier basis states (*n* ∈ [−100, 100] in eqs 2 and 4) and up to 20 nested commutators in truncated velocity gauge in the case of the bulk. For all of the bulk *t*PA spectra, we remove transition below 0.04 eV to account for the finite discretization of the Brillouin zone.

## Supplementary Material



## Data Availability

The data that support the findings of this study are available from the corresponding author upon reasonable request.

## Notes

Resolution of the nonequilibrium absorption spectra for the nanomaterial and bulk into individual transition lines; additional details demonstrating that the hybridization of pristine states leads to the broad absorption features in Figure 4 (PDF)
