# Supplementary material for: Transition from the Nanoscale to Bulk in the Nonequilibrium Optical Response of Laser-Dressed Materials
Source: J Phys Chem Lett. 2025 Dec 29;17(1):214–21. doi: 10.1021/acs.jpclett.5c02710 (PMC12794146; doi:10.1021/acs.jpclett.5c02710)
Supplement: Supplementary file 1 [file jz5c02710_si_001.pdf]

Supplementary Information: Transition from the Nanoscale to Bulk in the Non-equilibrium Optical Response of Laser-dressed Materials

Vishal Tiwari,[1] Luis Sierra-Ossa,[2] Pawel Wojcik,[3] and Ignacio Franco[1,4,5]

[1]*Department of Chemistry, University of Rochester, Rochester, New York 14627, USA*

[2]*Department of Chemistry, Northwestern University, Evanston, Illinois 60208, USA*

[3]*Department of Chemistry and Biochemistry, Florida State University, Tallahassee, Florida 32306, USA*

[4]*Department of Physics and Astronomy, University of Rochester, Rochester, New York 14627, USA*

[5]*Institute of Optics, University of Rochester, Rochester, New York 14627, USA*[*]

(Dated: November 26, 2025)

[*] ignacio.franco@rochester.edu

S1. ORIGIN OF THE SIZE-DEPENDENT LOW-FREQUENCY FEATURES

The strong dependence on chain length for the low-frequency features seen in Fig. 3 of the main text arises because the net effect is due to competing contributions that leads to a strong dependence on the effective density of states. This is demonstrated in Fig. S1 which plots the non-equilibrium absorption spectra along with the line spectra (vertical black lines) for bulk [Fig. S1(a)] and $N = 40$ [Fig. S1(b)] dressed with $E_\mathrm{d} = 1.0$ V/nm and $\hbar\Omega = 0.5$ eV. As seen, the overall spectra arises because of many possible contributions that do not exactly cancel one another.

As explained in the main text, the low-frequency features arise due to the formation and subsequent hybridization of the Floquet states in the material. The hybridization occurs when the Floquet states approximately coincide in energy. The number of possible hybridizations increase with the density of states in the material, and they all lead to low-frequency absorption or stimulated emission features. The net effect is the result of the competition between these contributions.

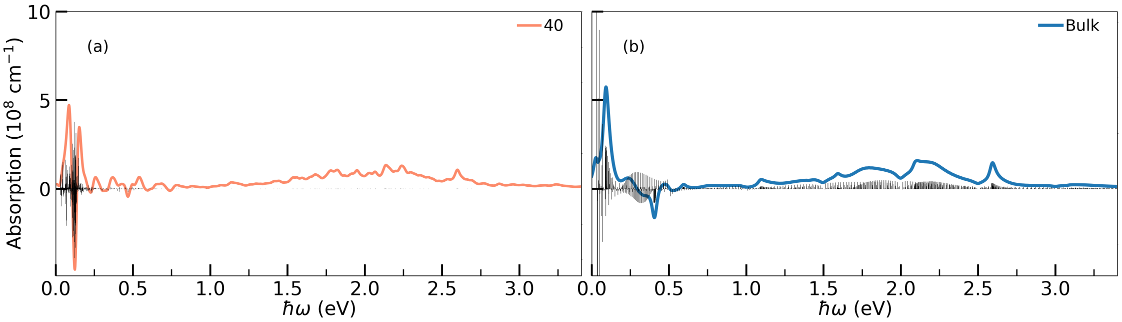

Fig. S1. Absorption spectra of the laser-dressed (a) bulk tPA and (b) nanomaterial with $N = 40$ unit cells for driving amplitude $E_\mathrm{d} = 1.0$ V/nm and $\hbar\Omega = 0.5$ eV. The line spectra shown as black lines in panels (a) and (b) represent the competing transition that do not exactly cancel giving rise to net absorption or stimulated emission.

S2. ORIGIN OF THE BROAD ABSORPTION FEATURES IN $N = 8$ NANOMATERIAL

As explained in the main text, the broad absorption features seen in Fig. 4(b) arise due to the hybridization of the pristine states leading to the emergence of multiple absorption

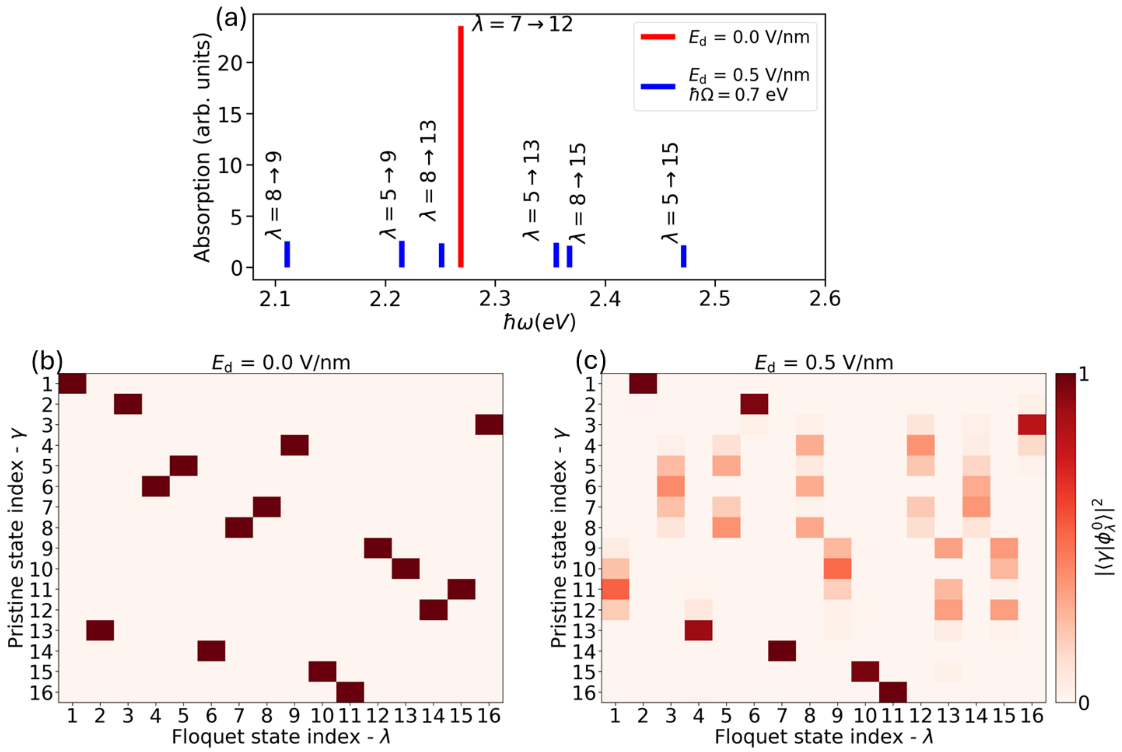

Fig. S2. (a) Transition lines in the absorption spectrum of the pristine (red) and laser-dressed (blue) $N = 8$ nanomaterial. Each transition is labeled by the contributing initial $\rightarrow$ final Floquet mode index λ. Projection $|\langle\gamma|\phi_\lambda(0)\rangle|^2$ of each of the pristine states γ in each of the Floquet modes λ for the (b) pristine and (c) laser-dressed nanomaterial. Floquet modes that are composed of only one of the pristine states in (b) now contain contributions from multiple pristine states due to laser-driving in (c). This leads to opening of new transition channels leading to multiple absorption peaks in (a).

features in the non-equilibrium optical response. Figure S2 explicitly shows this emergence of new transition lines and level hybridization in the laser-dressed $N = 8$ unit cell nanomaterial.

Figure S2(a) plots the individual transitions that occur in the $\hbar\omega \in [2.08, 2.6]$ eV range for the pristine (red) and laser-dressed nanomaterial (blue) with the same laser parameters as in Fig. 4. As seen the isolated absorption peak due to the HOMO $\rightarrow$ LUMO transition in pristine material at $\hbar\omega = 2.26$ eV is replaced by multiple absorption peaks when the nanomaterial is driven. To investigate the individual transitions and the involved Floquet states that lead to multiple absorption peaks in Fig. S2(a), we label each of the transition line with the contributing initial $\rightarrow$ final Floquet mode index λ.

The pristine states involved in the individual transitions between Floquet modes in Fig. S2(a) can be identified as follows. Each Floquet mode can be expanded in terms of the pristine material energy eigenstates $\{|\gamma\rangle\}$ as $|\phi_\lambda(t)\rangle = \sum_{n\gamma} F_{n\gamma}^{(\lambda)} e^{in\Omega t}|\gamma\rangle$ [1, 2], with $n \in \mathcal{Z}$. A projection of the Floquet modes at, say, time $t = 0$ into $|\gamma\rangle$, $|\langle\gamma|\phi_\lambda(0)\rangle|^2$, provides a way to identify the pristine material states contributing to each of the Floquet modes.

Figure S2(b)-(c) show such a decomposition for (b) $E_\mathrm{d} = 0.0$ and (c) $E_\mathrm{d} = 0.5$ V/nm. In the undriven case (b), individual Floquet modes have contribution from only one pristine material state. In particular, the HOMO ($\gamma = 8$) $\rightarrow$ LUMO ($\gamma = 9$) transition in Fig. S2(a) at $\hbar\omega = 2.26$ eV is due to a $7 \rightarrow 12$ transition between Floquet modes. By contrast, for the laser-driven case (c), each of the Floquet mode now has non-zero contributions from multiple pristine states. This is indicative of the mixing of pristine material states to form hybrid states.

Consider the transition from Floquet mode 8 to 9 in Fig. S2(a) at $\hbar\omega \sim 2.1$ eV for the laser-dressed nanomaterial. The Floquet mode 8 is now the linear combination of the pristine state $\gamma = 4$,6 and 8 while the Floquet mode 9 is that of pristine state 9,10 and 11. In this case, the LUMO is hybridizing with the neighboring LUMO+1 and LUMO+2 states while the HOMO with the HOMO-2 and HOMO-4 states because of the laser being resonant to their level spacing. Similarly, absorption line at $\hbar\omega = 2.21$ eV occurs due to transition from Floquet mode $5 \rightarrow 9$, where mode 5 is hybrid of 4,5,7 and 8 pristine material state (that is the HOMO, HOMO-1, HOMO-3 and HOMO-4). Similar analysis for other transitions concludes that the broad absorption peaks arising in laser-dressed nanomaterial occur due to the transition among hybrid Floquet modes formed from the mixing of neighboring pristine material states as discussed in the main text.

[1] B. Gu and I. Franco, Optical absorption properties of laser-driven matter, Phys. Rev. A **98**, 063412 (2018).

[2] H. Sambe, Steady states and quasienergies of a quantum-mechanical system in an oscillating field, Phys. Rev. A **7**, 2203 (1973).
